# Optimization of the method for the culture of melanocyte precursors from hair follicles and their activation by 1,25-dihydroxyvitamin D3

**DOI:** 10.3892/etm.2013.1252

**Published:** 2013-08-06

**Authors:** DAGUANG WANG, XIHUI XU, HUIJUN MA, XUEZHUANG YUE, CHENGRANG LI, WENYUAN ZHU

**Affiliations:** 1 Department of Dermatology, The First Affiliated Hospital of Nanjing Medical University, Nanjing, Jiangsu 210029;; 2 Department of Hematology, Nanjing Drum Tower Hospital, The Affiliated Hospital of Nanjing University Medical School, Nanjing, Jiangsu 210008, P.R. China

**Keywords:** melanocyte precursors, culture, 1,25 dihydroxyvitamin D3, activation

## Abstract

The melanocytes in vitiligo repigmentation are derived predominantly from melanocyte precursors (MPs) present in the outer root sheath (ORS) of hair follicles. The methods currently used for culturing MPs are unstable, and the cultured cells have the capacity to produce melanin. These factors are problematic when conducting *in vitro* studies to investigate the mechanism of repigmentation. Although 1,25-dihydroxyvitamin D3 (VID) has been demonstrated to be highly effective in the treatment of vitiligo in the clinic, its precise mode of action has yet to be elucidated. In the present study, the method for the culture of MPs from the ORS of hair follicles was optimized and the ability of VID to activate MPs was investigated. The results suggested that the MPs cultured using the optimized method mainly exhibited bipolar morphology. The cells proliferated well and were negative for 3,4-dihydroxy-L-phenylalanine (DOPA) staining. Transmission electron microscopy revealed that the cytoplasm of the MPs contained numerous stage I and stage II melanosomes; however, stage III and IV melanosomes were not observed. Following VID treatment, the MPs showed increased dendritic morphology, the cells stained positive for DOPA and stage III and IV melanosomes appeared in the cells. Western blotting revealed that microphthalmia-associated transcription factor (MITF), tyrosinase (TYR), tyrosinase-related protein-1 (TRP-1) and TRP-2 were expressed in the MPs and that VID increased the expression levels of MITF, TYR and TRP-1. However, the levels of MITF, TYR and TRP-1 in the MPs prior to and following VID treatment were significantly lower compared with those in cultured epidermal melanocytes, while the levels of TRP-2 in these three groups were not significantly different. Subsequent to VID treatment, the TYR activity in the MPs increased significantly, as did the corresponding melanin levels. In conclusion, the present study successfully optimized the method for MP culture. The MPs demonstrated no significant TYR activity or melanin synthesis; therefore, the MP cultures exhibited the features of MPs *in vivo*. In addition, VID significantly promoted the differentiation of MPs.

## Introduction

The melanocytes in vitiligo repigmentation are derived predominantly from melanocyte precursors (MPs) of the outer root sheath (ORS) of hair follicles (1). Thus, the *in vitro* culture of pure MPs is of value in the study of the mechanisms of vitiligo repigmentation. MPs were first successfully cultured by Tobin *et al* in 1995 (2); however, with the culture method that was utilized, it was difficult to avoid fibroblast contamination. In addition, the culture medium that was used promoted the differentiation and maturation of MPs. In a previous study, we improved the culture method of Tobin *et al* to reduce fibro-blast contamination; however, geneticin, which is frequently used in experiments to remove contamination, often leads to culture failure. In addition, the composition of our culture medium was the same as that used by Tobin *et al*, and although the cells were able to proliferate rapidly, they were not able to maintain the undifferentiated state (3). Further improvements to the composition of the culture medium have been reported; however, the cultured MPs continued to produce melanin (4). Cook *et al* successfully cultured human MPs established from neonatal foreskin in 2003 (5). Based on these studies, we optimized the method for the culture of MPs from hair follicles and performed MP identification analysis.

1,25-Dihydroxyvitamin D3 (VID) may be used for vitiligo treatment in the clinic; however, its mode of action has yet to be fully elucidated. In cellular models, VID has been shown to increase tyrosinase (TYR) expression in mouse MPs and promote melanin synthesis (6). VID has also been demonstrated to promote melanin synthesis in B16 melanoma cells (7). However, it has not been revealed whether VID functions as an activator of MPs from hair follicles. Therefore, in the present study, the activation effects of VID on cultured MPs were observed.

## Materials and methods

### Major reagents and materials

MCDB-153 culture medium and trypsin were purchased from Gibco-BRL (Grand Island, NY, USA). VID, stem cell factor (SCF), endothelin-3 (ET-3) and basic fibroblast growth factor (bFGF) were obtained from Peprotech, Inc. (Rocky Hill, NJ, USA), while Chelex-100, 3,4-dihydroxy-L-phenylalanine (DOPA), cholera toxin (CT) and dispase II were purchased from Sigma (St. Louis, MO, USA). Fetal bovine serum (FBS) was purchased from Hangzhou Sijiqing Biological Engineering Materials Co., Ltd. (Hangzhou, China) and the western blotting kits were obtained from Gene Company Ltd. (Shanghai, China). The antibodies for microphthalmia-associated transcription factor (MITF; 3F276), TYR (T311), TYR-related protein-1 (TRP-1; SPM456), TRP-2 (C-9) and glyceraldehyde 3-phosphate dehydrogenase (sc-59540) were all purchased from Santa Cruz Biotechnology, Inc. (Santa Cruz, CA, USA).

### Culture of MPs and melanocytes (MCs)

The study was approved by the Ethics Committee of The First Affiliated Hospital of Nanjing Medical University (Nanjing, China), and written consent was obtained from family members for the collection of scalp tissue from a cadaver (within 2 h of death). The donor was 21 years of age and the sample size was 6×5 cm^2^. The sample was disinfected with povidone-iodine and the galea aponeurotica was excised to the greatest extent possible. The scalp was sectioned into 0.3-cm-wide skin strips, and the strips were cut along the upper 0.1 cm of dermis. The region with epidermis was used for culturing MCs, while the area with dermis was used for culturing MPs. The method for the culture of the MPs was based on that described in our previous study (3), with certain modifications: i) Free follicles were obtained following digestion with 1% dispase II at 4°C for 24 h, instead of using collagenase; ii) melanoblast culture medium was used for the cell culture; and iii) a different passage method was used. Following 7 days of culture, a large number of cells had proliferated, including MPs. The first passage was conducted at a ratio of 3:1 (contents of 3 bottles collected into 1) and subsequent passages were conducted every 5–7 days at a ratio of 1:2 (contents of 1 bottle distributed into 2 bottles). Cells at the third passage consisted solely MPs and the cells at the fourth passage were used for experiments. The composition of the melanoblast culture medium used was as described by Cook *et al* (5). The basic medium comprised MCDB-153, 2 mM glutamine, 10% Chelex-100-chelated FBS, 2% FBS, 1.66 mg/l CT, 10 ng/ml SCF, 100 nM ET-3, 2.5 ng/ml bFGF, 50 U/ml penicillin and 50 g/ml streptomycin. The chelated serum was obtained by adding 15 g Chelex-100 to 500 ml serum and continuously mixing at 4°C for 1.5 h. MCs were cultured according to the method described by Cook *et al* (5), with the same culture medium as was used for the MPs.

### Experimental grouping

VID was prepared as a 10^−2^ M stock solution (using anhydrous ethanol as a solvent) and stored in the dark; the working solution was diluted using freshly prepared culture medium. The experimental samples were divided into four groups according to VID concentration: 0 (blank control), 10^−4^, 10^−6^ and 10^−8^ M. Following the treatment of the cells with VID for 72 h, the subsequent experiments were performed. The 10^−6^ M group was used for DOPA staining, transmission electron microscopy and western blotting. All four groups were used for the analysis of TYR activity and the measurement of melanin levels.

### DOPA staining

The control and MC groups and the 10^−6^ MVID MP group were used for this experiment. Following washing with phosphate-buffered saline (PBS), fixation with 2% paraformaldehyde and washing with PBS a further three times, the cells were incubated with 0.1% DOPA (dissolved in 0.1 M PBS) at 37°C for 5 h, prior to being counterstained with nuclear fast red.

### Transmission electron microscopy

The control and MC groups and the 10^−6^ M VID MP group were used for this experiment. Cells were dissociated using 0.25% trypsin and collected. Following fixation with 2.5% glutaraldehyde, cells were conventionally embedded and sectioned. The changes in the proportions of melanosomes were observed under a Hitachi H-7000 transmission electron microscope (Hitachi, Tokyo, Japan).

### Western blotting

The control and MC groups and the 10^−6^ M VID MP group were used for this experiment. Cells were dissociated using 0.25% trypsin and collected; total protein was then extracted and quantified using conventional methods. After denaturing, the total protein was subjected to 10% sodium dodecyl sulfate-polyacrylamide gel electrophoresis with 15 *μ*g protein in each lane. The protein bands were transferred onto an Immobilon-P transfer membrane (polyvinylidene fluoride). After blocking with 3% bovine serum albumin, the membrane was incubated with primary antibodies (3F276, T311, SPM456, C-9 and sc-59540) at a 1:1,000 dilution overnight at 4°C. After to washing, the membrane was incubated with secondary antibodies at room temperature for 1 h. The protein bands were developed using chemiluminescence reagents, and the gray-scale values were quantified using an image analysis system (Quantity One Software; Bio-Rad, Hercules, CA, USA).

### Detection of TYR activity and melanin level

The control group and each experimental group of MPs were used for this experiment. Cells were dissociated using 0.25% trypsin and counted. The TYR activity and melanin level were measured in accordance with previous studies (8,9). Briefly, the cells were treated with both control and VID for 72 h, washed, trypsinized and counted before pelleting. Melanin per cell was quantified after boiling in 1 M sodium hydroxide and melanin content in each sample was read from a calibration curve against synthetic eumelanin (Sigma) at 490 nm and converted to means ± SE melanin pg/cell from 3 independent experiments. For determination of TYR activity, the cells were washed in ice-cold PBS and were lysed with phosphate buffer (pH 6.8) containing 1% Triton X-100 and protease inhibitors (Complete™ protease inhibitor mixture; Roche Molecular Diagnostics, Basel, Switzerland). The lysates were clarified by centrifugation for 10 min at 10,000 × g. After the quantification of protein levels and adjusting concentrations using lysis buffer, 90 *μ*l of each lysate, containing an identical amount of protein, was placed in 96-well plates, and 10 *μ*l 15 mM L-DOPA was added to each well. After incubation at 37°C for 30 min, dopachrome formation was assayed by measuring absorbance at 475 nm using a microplate reader. TYR activity was reported as A475 values. Each experiment had three wells, and three independent experiments were conducted.

### Statistical analysis

All data are presented as the mean ± standard deviation. Data were compared using the paired t-test. The statistical analysis was performed using SPSS statistical software version 10.0 (SPSS, Inc., Chicago, IL, USA).

## Results

### Morphology of MPs

**Figure 1.**
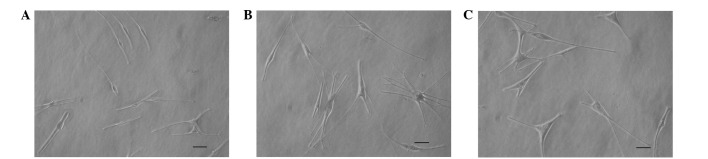
(A) Melanocyte precursors (MPs) in the control group are bipolar with small cell bodies and strong refractivity. (B) Following treatment with 1,25-dihydroxyvitamin D3 (VID) for 72 h, the cell bodies have increased in size and the dendrites extended, to provide cells with numerous levels of dendrites. (C) Cultured melanocytes are similar to VID-treated MPs, showing larger cell bodies and numerous dendrites (scale bar, 100 *μ*m).

The cultured MPs were bipolar cells with small, oval cell bodies and strong refractivity (Fig. 1A). Following VID treatment for 72 h, the cell bodies of the MPs increased in size and certain cells had extended dendrites and/or numerous dendrites (Fig. 1B). The cultured MCs were similar to VID-treated MPs, and they had larger cell bodies and numerous dendrites (Fig. 1C).

### DOPA staining and transmission electron microscopy

**Figure 2.**
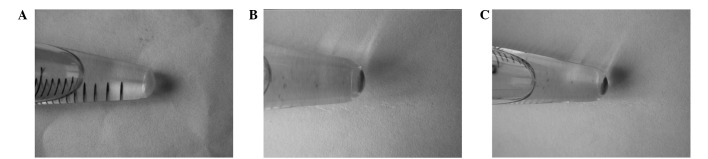
(A) The precipitate of melanocyte precursors (MPs) is yellow-white. (B) The precipitate of 1,25-dihydroxyvitamin D3 (VID)-treated MPs is gray-black. (C) The precipitate of melanocytes is similar to that of VID-treated MPs; however, the blackness is more apparent.

**Figure 3.**
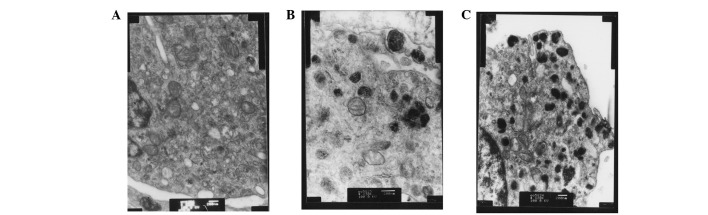
(A) Numerous stage I and II melanosomes are visible in the cytoplasm of the melanocyte precursors (MPs); however, no mature stage III or IV melanosomes are present. Melanosomes are predominantly distributed in the perinuclear region, and the mitochondria are regular without clear expansion. (B) Following treatment with 1,25-dihydroxyvitamin D3 (VID) for 72 h, there are numerous stage III and IV melanosomes in the cytoplasm. Mitochondria may be observed to have significantly proliferated and expanded. (C) The results in the melanocytes are similar to those in the VID-treated MPs: There are numerous stage III and IV melanosomes in the cytoplasm, and the melanosomes are distributed away from the nuclei and predominantly in the inner membrane (scale bar, 200 nm).

**Figure 4.**
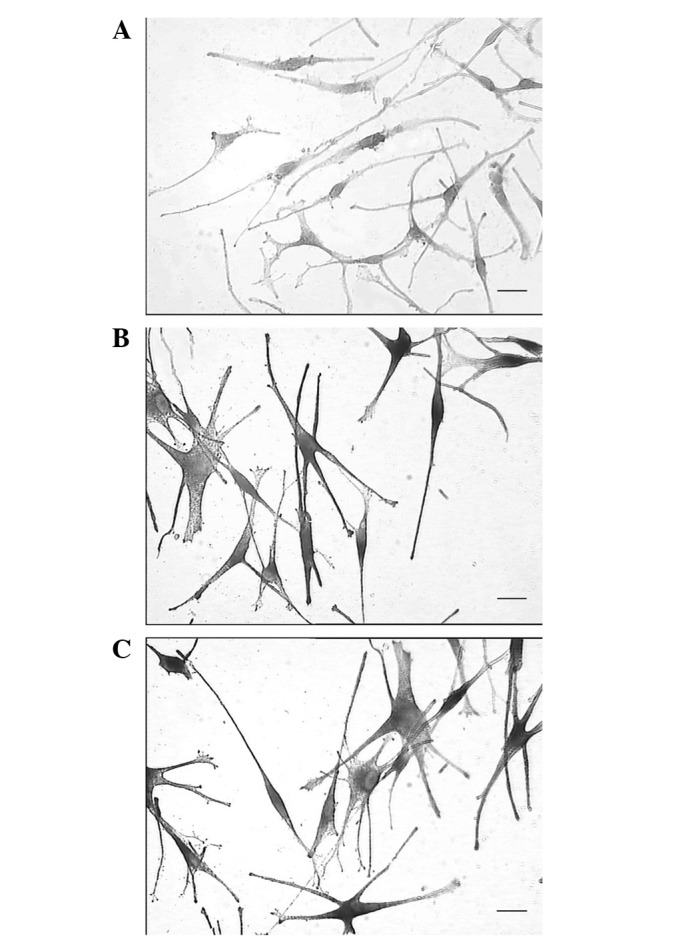
(A) No black granules are visible in the cytoplasm of the melanocyte precursors (MPs). The cytoplasm is stained by the nuclear fast red counterstain. (B) Following treatment with 1,25-dihydroxyvitamin D3 (VID), black granules are visible in the MPs, and the majority of the cells show strong 3,4-dihydroxy-L-phenylalanine (DOPA) staining, which obscures the nuclear fast red staining. (C) The results in the melanocytes are similar to those in the VID-treated MPs, showing strong positive staining and black granules that obscure the nuclear fast red staining. (DOPA stain, nuclear fast red counterstain; scale bar, 50 *μ*M.)

To further detect whether the MPs had matured, transmission electron microscopy was performed to observe the melanosomes, and DOPA staining was also performed to measure TYR activity. During cell collection, the cells were washed thoroughly with PBS five times to reduce the influence of impurities and the color of the medium on the precipitate. The results showed that the precipitate of the MPs was yellow-white (Fig. 2A), while the precipitate of the VID-treated MPs was gray-black, similar to the MCs (Fig. 2B and C). Observation under an electron microscope showed that the cytoplasm of the MPs contained numerous stage I and II melanosomes; however, no mature stage III or IV melanosomes were present. The melanosomes were predominantly distributed in the perinuclear region, and the mitochondria were regular without marked expansion (Fig. 3A). By contrast, VID treatment induced the appearance of several stage III and IV melanosomes in the cytoplasm of the MPs, similar to MCs. The location of the melanosomes in these cells was further away from the nuclei and predominantly in the inner membrane. In addition, the mitochondria in the VID-treated MPs were observed to have significantly proliferated and expanded, indicating that they were active (Fig. 3B and C). Therefore, these results suggested that the cultured MPs did not have melanin synthesis functions, that VID significantly activated the MPs and that the activated MPs had similar functions to MCs. The DOPA staining did not show black granules in the cytoplasm of the MPs, and the nuclear fast red counterstain showed a light-red result in the cytoplasm (Fig. 4A). Black granules became prominent in the cytoplasm of the VID-treated MPs, as well as in the MCs; certain cells showed strong positive staining, which covered the color of the nuclear fast red (Fig. 4B and C). Therefore, the DOPA stain results further indicated that the cultured MPs were in an undifferentiated state and that VID significantly promoted the differentiation of MPs into mature MCs.

### Western blotting

**Figure 5.**
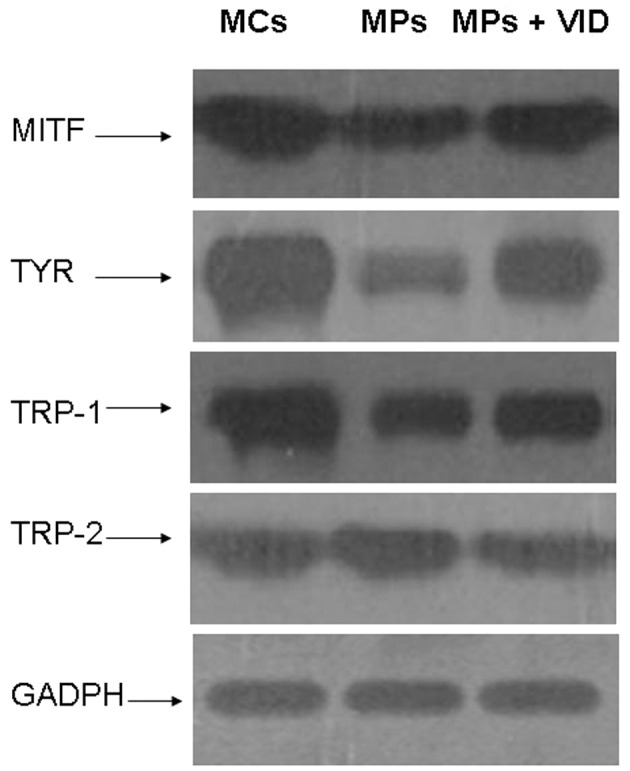
Expression levels of microphthalmia-associated transcription factor (MITF), tyrosinase (TYR) and TYR-related protein-1 (TRP-1) in melanocytes (MCs) are significantly higher than those in melanocyte precursors (MPs), while the level of TRP-2 is similar in these two cell types. Following treatment with 1,25-dihydroxyvitamin D3 (VID), the expression levels of MITF, TYR and TRP-1 are significantly increased in the MPs; however, they remain lower than those in the MCs. The expression of TRP-2 is not changed significantly with VID treatment. GADPH, glyceraldehyde 3-phosphate dehydrogenase.

The MPs and MCs expressed MITF, TYR, TRP-1 and TRP-2. The expression levels of MITF, TYR and TRP-1 in the MCs were higher than in the MPs, while the expression of TRP-2 in these two cell types was similar. Following VID treatment, the MITF, TYR and TRP-1 levels in the MPs significantly increased; however, they remained lower than those in the MCs. The expression of TRP-2 in the MPs was not significantly affected by VID (Fig. 5).

### Detection of TYR activity and melanin level

**Figure 6.**
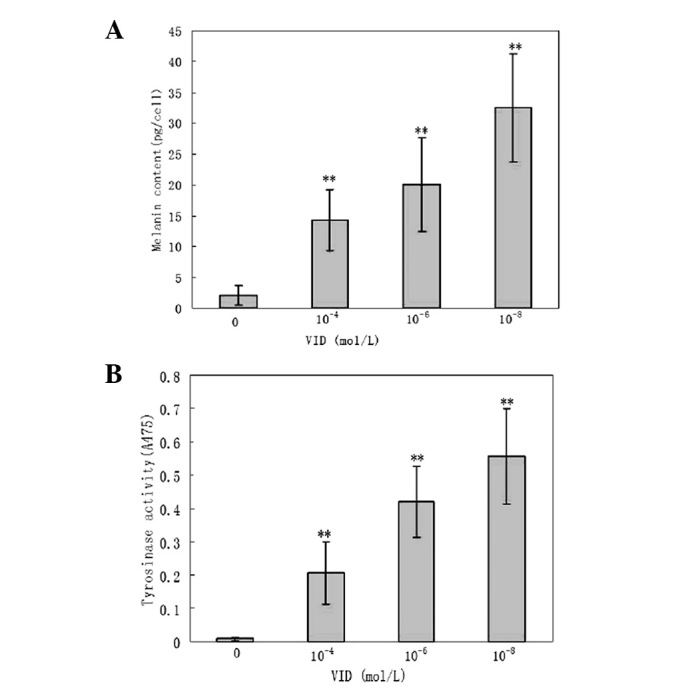
Treatment of melanocyte precursors (MPs) with different concentrations of 1,25-dihydroxyvitamin D3 (VID) promotes (A) tyrosinase (TYR) activity and (B) melanin synthesis in a concentration-dependent manner. The TYR activity is presented as the A475 value. Quantitation of melanin is based on the calibration lines constructed with a commercially available standard. n=9; ^**^P<0.01.

Compared with the control group, VID promoted TYR activity and melanin synthesis in MPs in a concentration-dependent manner (P<0.01; Fig. 6).

## Discussion

Numerous studies have demonstrated the successful culture of MPs (2–4). However, there are two main problems that remain unsolved: contamination by fibroblasts and, most importantly, the maintenance of the MPs in an undifferentiated state and without any melanin-synthesizing activity.

The fibroblasts that contaminate the MP culture mainly originate from the connective tissue sheath outside the ORS. Contamination may be reduced using two strategies: the first focusing on the separation of the hair follicles and the second focusing on the selection of the culture medium. During the separation of hair follicles, it is necessary to reduce the contamination with connective tissues to the minimum. In a previous study, we used dispase II combined with collagenase IV to separate the hair follicles, which greatly reduced connective tissue contamination; however, the experiments continued to fail occasionally due to contamination (3). Dispase II digests the basement membrane surrounding the hair follicles, reducing the adhesion of connective tissues to free hair follicles. Collagenase IV, however, digests the basement membrane and the dermis, thereby facilitating the adhesion of connective tissues to the free hair follicles. In the present study, we used 1% dispase II to digest the follicles for 24 h at 4°C, followed by 1 h at 37°C to eliminate the collagenase IV step. The results showed that this method had significant benefits: There was less adhesion between connective tissues and isolated hair follicles and no fibroblast contamination in the cultures, and the proliferation of cultured MPs was not blocked.

The second method of reducing fibroblast contamination entails using medium components that do not support the growth of fibroblasts. The growth of fibroblasts is Ca^2+^ dependent (10); thus, in order to reduce contamination, it is necessary to use a low-calcium culture medium. The culture medium used in the present study was MCDB-153. The difference between this medium and the Minimum Essential Medium (MEM)/RPMI-1640 media that have been used previously is that it has a lower Ca^2+^ concentration (RPMI-1640 medium: 0.42 mM; MCB-153: 0.03 nM). In addition, the FBS in the culture medium contributes Ca^2+^. In our experiment, the serum was reacted with the divalent metal ion-chelating agent, Chelex-100, prior to use, thereby reducing the increase in Ca^2+^ concentration caused by the serum. Due to the fact that some divalent cations are necessary for MP culture, 2% non-chelated serum was added to provide essential nutrients. Through these two modifications, it was observed that there was no fibroblast contamination in the culture and the MPs actively proliferated.

Since MPs only accounted for a small percentage of the confluent cells, the first passage during the culture process was performed at a 3:1 ratio. There were two advantages to this method: i) the MPs remained relatively highly concentrated, which was conducive to proliferation and ii) mature melanocytes died after passaging. Using these two features combined with the differential trypsinization method to remove keratinocytes, the percentage of MPs in the second generation reached 90%, while the mature melanocytes decreased in number and gradually died. Following 7–8 days, cells regained confluence and the passaging was conducted at the ratio of 1:2. There were no mature melanocytes or keratinocytes in the third generation of cells and there were only pure MPs.

The culture medium used in the present study was prepared according to the method described by Cook *et al* (5). The major additives were 10 ng/ml SCF, 100 nM ET-3, 2.5 ng/ml bFGF and 1.66 *μ*g/l CT. SCF is important in the survival, differentiation, proliferation and migration of MCs and appears to be required for MC culture. When binding to its ligand, c-kit, SCF is able to regulate the expression of MITF, thereby promoting MC proliferation and inhibiting melanin synthesis in MCs. Therefore, it is crucial in maintaining the undifferentiated state of MCs. Kawa *et al* (11) cultured the mouse melanocyte precursor cell line, NCC-4.1, and SCF was the only cytokine used to maintain cell proliferation and the undifferentiated state. ET-3 is also frequently used for melanoblast culture; it is necessary for the differentiation of neural crest cells into MCs and is able to significantly promote proliferation (10). The function of ET-3 in the differentiation of MPs remains inconclusive. It has been demonstrated that ET-3 is able to combine with SCF to promote the differentiation of neural crest cells into mature MCs (11). However, a different study showed that ET-3 was able to induce the dedifferentiation of MPs and transformation into glial cells (12). bFGF promotes MC proliferation, has mitogenic functions and has been used as an additive in a number of MP cultures. These cytokines are all able to significantly promote the proliferation of MCs and melanoblasts. However, they have bidirectional effects on cell differentiation: In certain conditions, they promote differentiation, while in other conditions, they inhibit differentiation. We hypothesized that these bidirectional effects on differentiation are predominantly associated with two factors: the status of the cells and the interaction of the components in the culture medium. With regard to the status of the cells, if MPs are at the neural crest cell stage, then all the previously mentioned factors promote differentiation; however, when the MPs are going to enter the mature melanocyte stage, SCF and ET-3 inhibit melanin synthesis. The other factor is the interaction of all components in the culture medium. The culture medium for the culture of epidermal melanoblasts used in a previous study (5) contained four commonly used proliferation-promoting agents: SCF, ET-3, bFGF and CT. Those four components cooperated to not only promote proliferation but also to maintain cells in an immature state. By applying this method, we successfully cultured MPs from hair follicles.

The DOPA staining of MCs *in vivo* is positive, indicating that MCs have the ability to synthesize melanin, while the DOPA staining of MPs is negative, indicating that MPs have no melanin synthesizing ability (13). In the present study, under an electron microscope, stage III and IV melanosomes were observed in MCs while MPs contained only stage I and II melanosomes in the absence of VID. The methods used to culture MPs from hair follicles in previous studies all promoted melanin synthesis in MPs (2–4). Therefore, although those cultured MPs were different from epidermal melanocytes, they had functionally transformed into mature melanocytes. The cultured MPs in the present study were DOPA-negative and did not contain melanosomes above stage II under an electron microscope, indicating that they were in the MP state and were closer to true MPs *in vivo* (13,14). MPs express MITF, TRP-2 and TRP-1 but not TYR *in vivo*. The present western blotting results showed that the cultured MPs expressed MITF, TRP-2, TRP-1 and TYR. The expression levels of TRP-2 in the MPs and MCs were not significantly different; by contrast, the expression levels of MITF, TRP-1 and TYR in the MPs were significantly lower than those in the MCs. Due to the fact that epidermal MCs and MPs used the same culture medium, these results further indicated that the cultured MPs and MCs were biologically distinct, despite the fact that the MPs showed some differentiation.

The topical treatment of vitiligo using VID in clinical practice produces excellent results. However, its mechanism of action has not been studied in depth. The repigmentation of vitiligo is mainly dependent on the activation of MPs in the ORS of hair follicles and their migration to the epidermis (1). Therefore, an aim of this study was to confirm whether VID had an activating effect on MPs derived from the ORS of hair follicles. The results showed that VID promoted dendrite formation in the MPs, increased cell body size, increased the expression of enzymes involved in melanin synthesis and catalyzed melanin synthesis. Electron microscopy and chemical detection methods demonstrated that VID promoted melanin synthesis in MPs.

VID is able to increase TYR activity in B16 melanoma cells and promote melanin synthesis (7); however, it is still controversial whether VID is able to promote melanin synthesis in normal epidermal melanocytes. Mansur *et al* (15) showed that VID did not affect melanin synthesis in MCs (15); however, Tomita *et al* (16) demonstrated that VID promoted TYR expression in melanocytes (16). VID is able to significantly induce the differentiation of mouse MPs (6); this was further confirmed by the present results. Abdel-Malek *et al* (17) reported that the topical application of VID increased the number of DOPA-positive melanocytes in the epidermis (17). Therefore, based on these previous and current results, we propose that the mechanism by which VID counters vitiligo involves the activation of MPs from the ORS, followed by the entry of the MPs into the epidermis and their differentiation into MCs.
